# Inferring Resilience to Fragmentation-Induced Changes in Plant Communities in a Semi-Arid Mediterranean Ecosystem

**DOI:** 10.1371/journal.pone.0118837

**Published:** 2015-03-19

**Authors:** Ángel de Frutos, Teresa Navarro, Yolanda Pueyo, Concepción L. Alados

**Affiliations:** 1 Pyrenean Institute of Ecology (CSIC), Jaca (Huesca), Spain; 2 Pyrenean Institute of Ecology (CSIC), Zaragoza, Spain; 3 Department of Vegetal Biology, Málaga University, Málaga, Spain; Institute of Ecology and Biodiversity, CHILE

## Abstract

Predicting the capacity of ecosystems to absorb impacts from disturbance events (resilience), including land-use intensification and landscape fragmentation, is challenging in the face of global change. Little is known about the impacts of fragmentation on ecosystem functioning from a multi-dimensional perspective (multiple traits). This study used 58 500-m linear transects to quantify changes in the functional composition and resilience of vascular plant communities in response to an increase in landscape fragmentation in 18 natural scrubland fragments embedded within a matrix of abandoned crop fields in Cabo de Gata-Níjar Natural Park, Almería, Spain. Changes in functional community composition were measured using functional diversity indices (functional richness and functional dispersion) that were based on 12 plant traits. Resilience was evaluated using the functional redundancy and response diversity from the perspective of plant dispersal, which is important, particularly, in fragmented landscapes. Scrubland fragmentation was measured using the Integral Index of Connectivity (IIC). The functional richness of the plant communities was higher in the most fragmented scrubland. Conversely, the functional dispersion (i.e., spread) of trait values among species in the functional trait space was lower at the most fragmented sites; consequently, the ecological tolerance of the vegetation to scrubland fragmentation decreased. Classifying the plant species into four functional groups indicated that fragmentation favoured an increase in functional redundancy in the ‘short basal annual forbs and perennial forbs’ group, most of which are species adapted to degraded soils. An assessment based on the traits associated with plant dispersal indicated that the resilience of ‘woody plants’, an important component in the Mediterranean scrubland, and habitat fragmentation were negatively correlated; however, the correlation was positive in the ‘short basal annual forbs and perennial forbs’ and the ‘grasses’ groups.

## Introduction

Predicting changes in ecosystem resilience to environmental stress is an important subject in ecological research [1] because the changes can increase the vulnerability of an ecosystem to disturbance events (e.g., fire, grazing, land-use intensification, habitat fragmentation). Often, ecosystem resilience is defined as the ability of its constituent species to tolerate such events and thereby allow the ecosystem to maintain or recover its functions and processes [2], [3]. However, resilience has been defined and interpreted in various ways [4]. Consequently, the methods used to evaluate resilience have differed depending on the context in which it has been assessed. Recent studies have used the concept of ecosystem functioning to assess ecosystem resilience [1,5]. In that framework, the resilience in ecosystem functioning can be eroded by, for instance, land-use intensification [6], which modifies the landscape and can affect the amount of habitats or other resources that are required by species. The reduction in ecosystem resilience is a result of environmental filtering of the regional species pool [7], which favours species that have specific functional traits (i.e., morpho-physio-phenological features that affect fitness and are measurable at the individual level [3], [8]) well-adapted to habitat changes [9]. Thus, functional diversity provides a mechanistic link between ecosystem resilience and species [1].

Functional diversity measures biodiversity from a functional perspective based on species’ traits [10], [11]. Recently, various approaches have been used to study ecosystem functional diversity [3], [6], [12–14] that includes a variety of single- and multi-trait indices for measuring functional diversity [12], [15–17]. Recent studies have found correlations between multi-dimensional indices (i.e., multi-trait indices) and several complex properties of ecosystems such as niche differentiation [18], [19] and ecosystem resilience [6], [5], [20], but see [21]. For example, some multi-trait indices (e.g., index of functional dispersion) measure the diversity of responses to disturbances mediated by the functional traits of the organisms present in a given ecosystem [6]. An increase in the variety of functional responses to a disturbance among species that perform similar functions can increase the resilience of the ecosystem [5], [22]. Multi-trait indices can quantify the change in functional community composition across gradients of environmental stress [14], [18] such as eutrophication [23], grazing [24], land-use intensification [6], [25], forest degradation [26], and habitat fragmentation [27]. For example, Laliberté *et al*.[6] demonstrated that an increase in land-use intensification reduced the response diversity in plant communities, which reduced ecosystem resilience.

Humans have been responsible for most of the habitat fragmentation, which is widely recognized as a major threat to biodiversity, globally [28], [29], and has involved landscape changes such as natural habitat loss, fragment isolation, and reductions in fragment size and habitat quality [30], [31]. Fragmentation affects taxonomic diversity, which can lead to local species extinctions and disrupt the functioning of ecosystems [29], [32–34]. For instance, Alados *et al*. [35] demonstrated that severe scrubland fragmentation disables important mechanisms such as spatial self-organization, facilitation, and plant dispersal, which results in a reduction in species diversity. Furthermore, habitat fragmentation leads to the transformation of ecosystems into ones that have functionalities that differ from those of the original. This can affect community resilience after disturbance, and can have important implications for conservation biology [32], [36]. A better understanding of the effects of fragmentation on community functional diversity and ecosystem resilience is essential for designing of effective conservation strategies. To our knowledge, no studies have used a multi-dimensional functional perspective to assess changes in the functional composition and resilience of plant communities in landscapes altered by habitat fragmentation.

In this study, we assessed the changes in functional composition and resilience of the plant communities in response to habitat fragmentation within a well-preserved Mediterranean scrubland in the Cabo de Gata-Níjar Natural Park (Spain). This Natural Park is part of an important biodiversity hotspot and is important for biological conservation in the region [37]. In arid and semi-arid areas, ecosystem resilience is important because disturbances can lead to irreversible ecological degradation (e.g., [38–41]), a subject of considerable theoretical studies [39], [40], [42], [43]. In our study, we used multi-dimensional functional diversity to assess the functional composition and resilience of plant communities in a semi-arid ecosystem. Specifically, we measured various types of multi-dimensional functional diversity that assess the functional composition and resilience of the plant communities across a gradient of scrubland fragmentation. First, to assess the response of community functional diversity to fragmentation, we measured changes in functional richness (i.e., the amount of functional trait space filled by all species in the community [12]) and functional dispersion (i.e., the spread of trait values in the functional trait space occupied by the community [15]) in the plant communities. Fragmentation can lead to habitat loss and degradation; therefore, we predicted that the functional richness and functional dispersion in the plant communities will be lower in the most fragmented sites. Second, to assess the vegetation resilience to fragmentation, we used functional redundancy and response diversity as proxies of resilience, which have to be evaluated concurrently [1]. Functional redundancy is the number of species within each functional group that have similar ecological effects on ecosystem functioning, based on effect traits (i.e., functional effect groups; sensu [10], [6]). Habitat fragmentation can have detrimental effects on species richness; therefore, we predicted that functional redundancy will be lower in the most fragmented sites. Finally, we assessed whether species within each functional effect group responded differently (“response diversity”; sensu [22]) to habitat fragmentation based on the plant dispersal mechanism (i.e., using traits associated with plant dispersal). The plant dispersal mechanism is sensitive to habitat fragmentation [35]; therefore, we predicted that response diversity will be lower in the most fragmented sites.

## Materials and Methods

### Study area

The study area was in Cabo de Gata-Níjar Natural Park (49,512 ha, including 12,012 marine ha [44]) in the province of Almería, SE Spain (park centered at 2°4’W 36°52’N). The study area was in the volcanic portion of the Park (for details, see [45]), where the climate is semi-arid Mediterranean, the annual average temperature is 19.4°C, and the mean annual rainfall is 193.9 mm. Elevation ranges from sea level (coastal areas) to 493 m (El Fraile Peak). The rural population is small (<5,500 inhabitants in 2008 [46]) and farming has been based on traditional agro-pastoral systems [44]. Dry arable cereal farms have replaced natural scrubland, except on the hills. Consequently, natural scrubland within the study area occurs in patches of various sizes. Since the 1960s, most of the arable lands have been abandoned because of low yields and have converted to arid garrigue [45], which were excluded from our study; rather, we surveyed the areas that had natural scrubland dominated by *Chamaerops humilis*, *Rhamnus lycioides*, *Pistacia lentiscus*, and *Periploca laevigata* [47]. Sheep and goats lightly grazed the natural scrubland and the stocking rate has been < 0.5 livestock units per hectare [48]. That said, areas of moderately or highly-grazed natural scrubland were excluded from the study.

### Plant surveys and plant traits

In April 2006, we surveyed the vegetation in 18 natural scrubland fragments that differed in size (range = 44.1–3,308.5 ha) and distance between them (range = 43.6–10,690.8 m), but had similar soils [45]. Within each fragment, 2–4 500-m linear transects were established (58 in total) oblique to the slope and along different, randomly selected angles, which prevented anisotropy. To record the presence of individual plants and species in each transect, we used the Point-intercept method (contact points every 20 cm [49]). For analytical purposes, we included species that had at least five occurrences within each transect in at least one fragment (namely, 94 from 306 plant species detected, which was 96.5% of the vegetation cover; see S1 Table). Botanical nomenclature followed Castroviejo *et al*. [50]. Based on our experience in semi-arid ecosystems and the data available, to capture the characteristics known to be important in fragmented landscapes, we measured 12 functional plant traits, which were assumed to reflect the sensitivity of plant species to scrubland fragmentation [35], [51], [52]. In 2005 and 2006, the 12 traits were measured in 94 plant species (10–20 individuals per species collected throughout the study area; Table 1; see details in [51]). For each individual, the following traits were measured in the laboratory: plant height, seed mass, seed number, clonality, potential spatial dispersal, alternative spatial dispersal (i.e., presence or absence of secondary spatial dispersal), and pollination syndrome traits. For the other traits (i.e., growth form, main growth form, life cycle, biological type, and plant architecture; see references in Table 1), we used published information. The 12 traits were categorical or ordinal (Table 1). To avoid correlations among traits, traits were not derived from other traits (S2 Table [12]). All plant surveys and trait measurements were performed under permits issued by the Delegación Provincial de la Consejería de Medio Ambiente de la Junta de Andalucía, Spain. The plant trait data are available from the TRY Database (http://www.trydb.org/TryWeb/Home.php). Environmental data of plant surveys and species abundance data are presented in S1 Appendix and S2 Appendix, respectively.

**Table 1:** Plant traits used in the analyses of the plant communities.

Plant trait	Description	Trait type	E/R	Reference
Growth Form	1: Cushion; 2: Dwarf shrubs; 3: Erect leafy; 4: Leafless; 5: Non tussock grass; 6: Palmoid; 7: Short basal; 8: Shrub; 9: Small shrub; 10: Tree; 11: Tussock grass	Categorical trait	E	Cornelissen *et al*. (2003)
Main Growth Form	1: Grass; 2: Forb; 3: Woody species	Categorical trait	E	Cornelissen *et al*. (2003)
Life cycle	1: Annual; 2: Perennial species	Categorical trait	E	Blanca *et al*. (2009)
Clonality	1: Absent; 2: Present	Categorical trait	R	Blanca *et al*. (2009)
Plant height	1: <10 cm; 2: 11–29 cm, 3: 30–59 cm; 4: 60–99 cm; 5: 1–3 m	Ordinal trait	E	Navarro *et al*. (2009a)
Seed mass	1: 0.01–0.099 mg; 2: 0.1–0.999 mg; 3: 1–9.999 mg; 4: 10–99,999 mg; 5: 100–1000 mg	Ordinal trait	R	Navarro *et al*. (2009a)
Seed number	1: 0–250; 2: 251–500, 3: 501–1000; 4: 1001–2500; 5: 2501–5000; 6: >5001	Ordinal trait	R	Navarro *et al*. (2009a)
Potential spatial dispersal	1: Developed spatial dispersal by abiotic vectors; 2: Developed spatial dispersal by biotic vectors; 3: Restricted spatial dispersal	Categorical trait	R	Navarro *et al*. (2009a)
Alternative spatial dispersal	1: Absent; 2: Present	Categorical trait	R	Navarro *et al*. (2009a)
Biological type	1: Chamaephyte-caespitose; 2: Chamaephyte-fruticose; 3: Chamaephyte-pulvinular; 4: Chamaephyte-creeping; 5: Chamaephyte-succulent; 6: Chamaephyte-sufruticose; 7: Geophyte-rhizomatous; 8: Hemicryptophyte- caespitose; 9: Hemicryptophyte-erect; 10: Hemicryptophyte-escapiform; 11: Hemicryptophyte-creeping; 12: Hemicryptophyte-rosulate; 13: Nano-phanerophyte-genistoid; 14: Nano-phanerophyte-evergreen; 15: Nano-phanerophyte-pulvinular; 16: Nano-phanerophyte-climber; 17: Therophyte-caespitose; 18: Therophyte-erect; 19: Therophyte-creeping; 20: Therophyte-rosulate	Categorical trait	E	Blanca *et al*. (2009)
Pollination syndrome	1: Anemophily; 2: Entomophily	Categorical trait	E	Navarro *et al*. (2009a)
Plant architecture	1: Champagnat †; 2: Corner **; 3: Corner †; 4: Holttum **; 5: Holttum †; 6:Leeuwenberg †; 7: Rauh *; 8:Scarrone ***; 9: Scarrone +; 10: Scarrone †	Categorical trait	E	Navarro *et al*. (2009b)

Plant traits used in the analyses of the plant communities in Cabo de Gata-Níjar Natural Park, Spain. These 12 plant traits were used to assess the overall plant community response to fragmentation. Functional effect groups were classified based on seven effect traits (E). Mechanisms of plant dispersal were assessed based on the following response functional traits (R): seed mass, seed number, potential spatial dispersal, and alternative spatial dispersal (i.e., high possibility of dispersal by two or multiple agents or vectors). Note that ‘Alternative spatial dispersal’ is presence or absence of secondary spatial dispersal. Symbols in ‘Plant architecture’ indicate the architectural model (see reference [52]).

### Response of functional community composition to scrubland fragmentation

To assess the response in the functional composition of the plant communities to fragmentation, we quantified the changes in functional richness and functional dispersion in the plant communities across a gradient in the degree of scrubland fragmentation, following the methods of Laliberté *et al*. [15] and Villéger *et al*. [12]. Those indices measure the effects of disturbance on functional biodiversity. For example, Komac et al. [53] showed that grazing increases functional richness and functional dispersion in sub-alpine and alpine grasslands, which indicated that grazing was an essential mechanism in structuring these grasslands. In our study, functional richness was quantified using the multivariate FRic Index [12], in which all plant traits (n = 12) were categorical or ordinal variables (Table 1); thus, functional richness was the number of unique trait combinations in the plant community [54]. The relative abundance of species does not affect functional richness. Functional dispersion was quantified using the multivariate FDis Index [15], [54], which measures the average distance of each species from the centroid in the functional trait space. To compute distances, we used Gower’s Dissimilarity because it is suited to categorical traits [15]. The FDis Index incorporates the relative abundance of species and is not strongly influenced by outliers. Communities that have a high dispersion of species in the trait space (i.e., high functional dispersion) reflect the high degree of trait dissimilarity among species, depending on traits used. Functional richness and functional dispersion were not correlated (Spearman’s rank correlation: |r_s_| = 0.1).

### Vegetation resilience to scrubland fragmentation

Ecosystem resilience was assessed by quantifying two aspects of functional diversity that contribute to resilience (i.e., functional redundancy and response diversity [1]). To assess the effects of scrubland fragmentation on functional redundancy and response diversity, we performed a functional classification of the plant species, which followed the hierarchical effect—response functional trait framework [6], [10], [24]. Following the recommendations of Cornelissen *et al*. [55] and Laliberté *et al*. [6], the functional traits were classified as either an effect or a response trait (see Table 1). Functional effect traits influence ecosystem functioning, and functional response traits affect plant responses to environmental drivers [6], [11], [20], [24] such as disturbances (in our study, scrubland fragmentation). Plant species were assigned to functional effect groups (plant species that have similar ecological effects) based on functional effect traits, and Gower’s Dissimilarity Distance and Ward’s Clustering method [6], [56]. According to our experience in semi-arid ecosystems, in our dataset of 12 traits, seven traits were effect traits because they are likely to contribute to ecosystem functioning in these semi-arid ecosystems (see references in Table 1). In a post-hoc examination of the clusters, we identified the functional effect groups based on expert botanical knowledge [6]. The number of species in each functional effect group was the measure of functional redundancy [6], [54]; that is, the functional effect groups that have the most species have the highest functional redundancy. In addition, resilience might be higher if species within a functional effect group respond differently to disturbance because of the response traits associated with a particular disturbance (i.e., “response diversity”; sensu [22]). Therefore, we assessed the response diversity in each functional effect group for the function of plant dispersal, which is an important mechanism in fragmented landscapes [35]. To that end, for each functional effect group, we calculated the response diversity using the FDis Index based on the functional traits directly associated with the dispersal function (i.e., four functional response traits; see Table 1; see [5], [6]), which takes into account the relative abundance of species.

### Scrubland fragmentation

To quantify scrubland fragmentation, we used a landscape connectivity metric: the Integral Index of Connectivity (IIC [57]). IIC has the properties required of a network metric used in fragmented landscapes, and has been recommended for conservation purposes [58], and assessing ecosystem changes. IIC quantifies overall landscape connectivity by integrating patch size (in our case, size of the natural scrubland fragments) and connectivity between patches (distances between natural scrubland fragments). The relative contribution (dIIC) of each natural scrubland fragment to overall IIC was calculated as

$$dIIC_{i}=100\times \;\frac{IIC-\;IIC_{remove,\;\;i}\;}{IIC\;}$$

where *dIIC*_*i*_ is the relative (%) contribution of fragment *i* in the change in *IIC* that would occur if fragment *i* was removed from the study area [57]. For that, we estimated the sizes of all natural scrubland fragments (n = 269 fragments) and the distances between them (mean distance ± SD = 10.83 ± 8.65 km) within the study area based on a digitized map of natural scrubland fragments [45]. For statistical analyses, we used the dIIC values (%) of each scrubland fragment surveyed (n = 18, range = 44.1–3,308.5 ha) as a measure of the contribution to overall IIC, which was calculated using Conefor Sensinode software [59]. The range of dIIC values reflected the gradient in scrubland fragmentation, and low dIIC indicates a small contribution by the scrubland fragment in maintaining overall landscape connectivity (i.e., highly fragmented scrubland).

In addition, winds that contain moist marine air (e.g., sea breezes) can influence plant communities within the study area, which is one of the most arid regions in Europe. For that reason, we measured exposure (facing or not facing the sea) and distance to the sea (range: 0.1–6.8 km) of each plant community using ArcGIS software by ESRI.

### Statistical analyses

To assess the response in functional composition and resilience to fragmentation in the plant communities, we used Generalized Linear Mixed Models (GLMM [60]), which accommodated an uneven number of pseudo-replicated transects per fragment (range: 2–4 pseudo-replicates) and included fragment as random effect. The GLMM residuals approximated a Poisson distribution when either functional richness or functional redundancy was included as a response variable. However, the residuals approximated a normal distribution when either functional dispersion or response diversity was included as a response variable. dIIC, exposure, and distance to sea were included as explanatory variables, which were centred to alleviate correlations between fixed effects [60]. In addition, the quadratic terms of the continuous explanatory variables were assessed. In the analysis based on functional groups, functional effect group (categorical variable) was included as an explanatory variable in the GLMM for either functional redundancy or response diversity. In those models, to identify the combinations of levels (i.e., interaction terms) that differed from each other, we used post-hoc comparisons. To identify the best models, we followed the recommendations of Zuur *et al*. [60] for selecting a mixed-effect model and used Akaike’s Information Criterion (AIC). All models were validated by verifying (i) the homogeneity between model residuals versus fitted values, (ii) the histogram of the model residuals for normality, and (iii) the absence of spatial auto-correlation in the residual [60], [61]. All GLMM were performed using the ‘nlme’ [62] and ‘lme4’ [63] packages in the R environment [64] using the Restricted Maximum-Likelihood Estimation Method (REML), which produces unbiased estimates of model parameters [60]. All interaction model plots were performed using the ‘effects’ R package [65].

## Results

### Response of functional community composition to scrubland fragmentation

**Fig 1:**
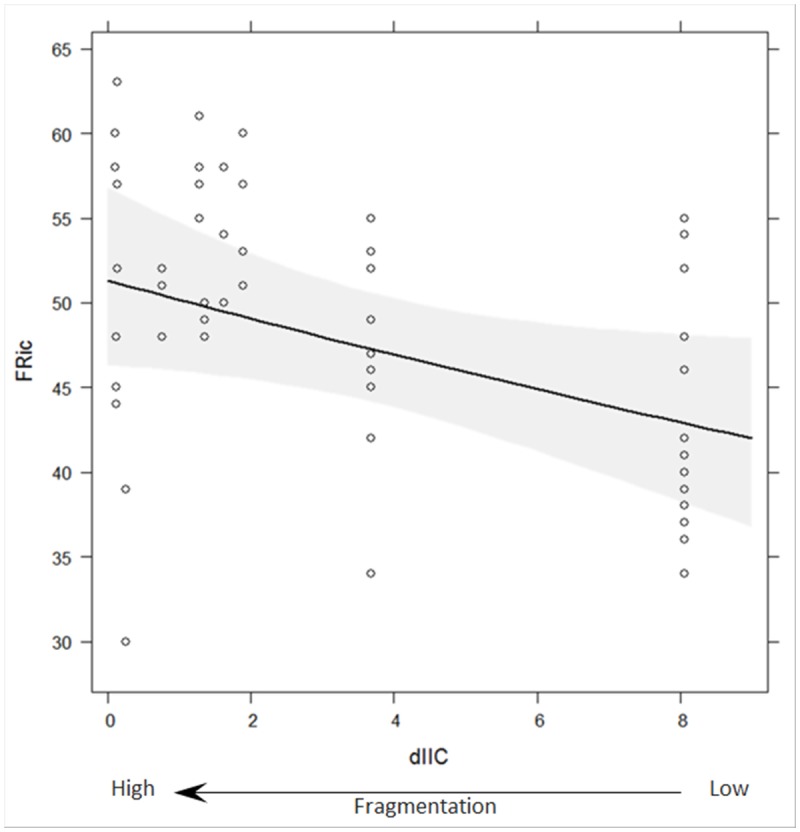
Functional richness in plant communities across a scrubland fragmentation gradient. Fitted values (solid line) and 95% confidence band (grey band) for the optimal Poisson GLMM model applied to the observed functional richness (FRic) in plant communities across a scrubland fragmentation gradient (dIIC) in Cabo de Gata-Níjar Natural Park, Spain. dIIC (here, square-root transformed) is the relative contribution (%) of each scrubland fragment surveyed to overall landscape connectivity. Low dIIC indicates high fragmentation.

In Cabo de Gata-Níjar Natural Park (SE Spain), the functional richness in the plant communities and scrubland fragmentation were significantly positively associated (p = 0.0378; Fig. 1); i.e., the most fragmented sites had the most functionally diverse plant communities (based on the best GLMM). In the model, exposure to and distance to sea were not statistically significant predictors; i.e., exposure to sea did not have a significant effect on the functional richness in the plant communities.

**Fig 2:**
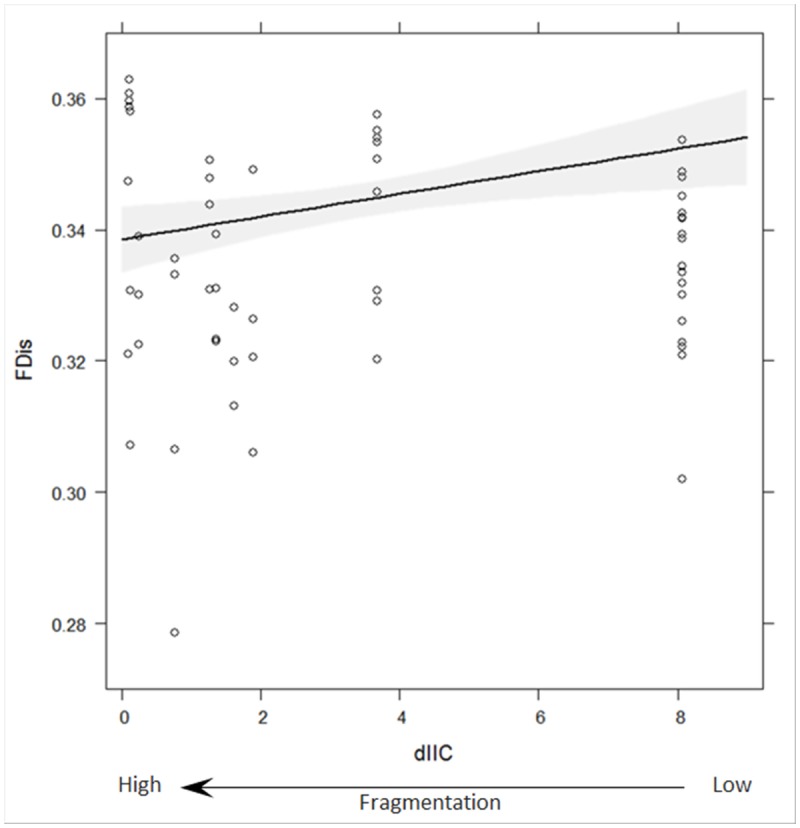
Functional dispersion in plant communities across a scrubland fragmentation gradient. Fitted values (solid line) and 95% confidence band (grey band) for the optimal Gaussian GLMM model applied to the observed functional dispersion (FDis), weighted by relative abundance of species, in plant communities across a scrubland fragmentation gradient (dIIC) in Cabo de Gata-Níjar Natural Park, Spain. dIIC (here, square-root transformed) is the relative contribution (%) of each scrubland fragment surveyed to overall landscape connectivity. Low dIIC indicates high fragmentation.

The best GLMM for functional dispersion in the plant communities included the terms dIIC, exposure to sea, distance to sea, and the interaction term dIIC x exposure to sea, which indicated that scrubland fragmentation significantly reduced the functional dispersion in the plant communities (p = 0.0001; Fig. 2). In that model, the exposure to sea term was not significant; however, the interaction effect with dIIC was negative and significant, which indicated that the functional dispersion in the plant communities was highest at the least fragmented sites that were not exposed to the sea (p <0.0001; S1 Fig.). In that model, the distance to sea term was positive and significant, which indicated that the functional dispersion in the plant communities increased as the distance to the sea increased (p <0.0001; S2 Fig.).

### Vegetation resilience to scrubland fragmentation

Four functional effect groups were identified in the clustering structure based on seven effect traits (Table 1); however, primarily, two traits (Main Growth Form and Growth Form, Table 1) differentiated the four functional effect groups [(1) ‘woody plants’, (2) ‘erect annual forbs’, (3) ‘short basal annual forbs and perennial forbs’, and (4) ‘grasses’; S3 Table].

**Fig 3:**
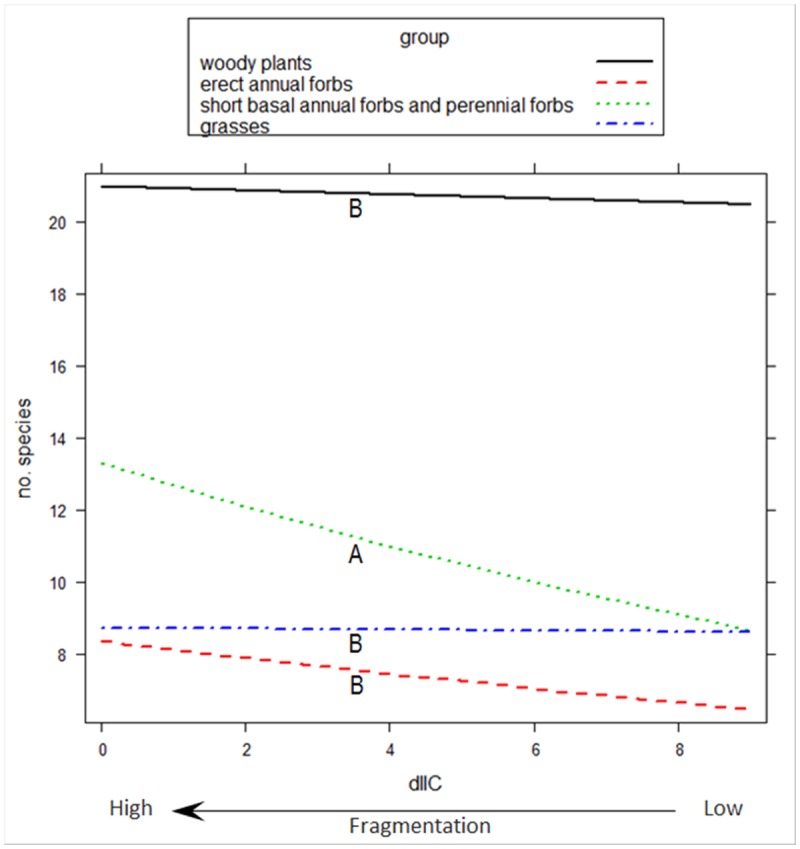
Functional redundancy of four functional effect groups across a scrubland fragmentation gradient. Functional redundancy (no. of species) of four functional effect groups across a scrubland fragmentation gradient (dIIC) in Cabo de Gata-Níjar Natural Park, Spain. dIIC (here, square-root transformed) is the relative contribution (%) of each scrubland fragment surveyed to overall landscape connectivity. Low dIIC indicates high fragmentation. For clarity, the observed functional redundancy points are omitted. Group 1: woody plants; Group 2: Erect annual forbs; Group 3: short basal annual forbs and perennial forbs; and Group 4: grasses. Different letters below each line indicate statistically significant differences in species number with fragmentation across groups based on GLMM followed by multiple pairwise comparisons.

**Fig 4:**
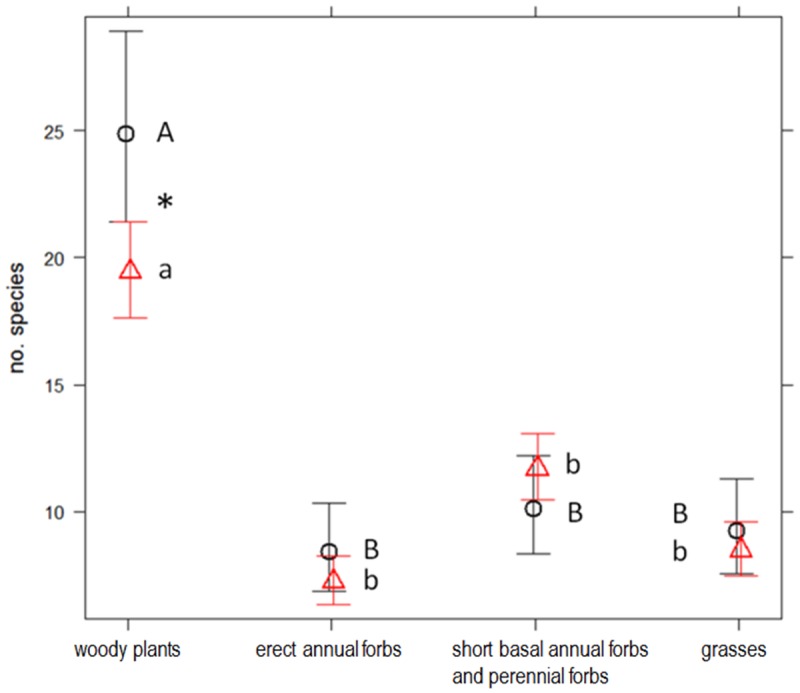
Average functional redundancy of four functional effect groups at non-sea-facing and sea-facing sites. Average functional redundancy (no. of species) of four functional effect groups at non-sea-facing (circles) and sea-facing (triangles) sites in Cabo de Gata-Níjar Natural Park, Spain. Group 1: woody plants; Group 2: Erect annual forbs; Group 3: short basal annual forbs and perennial forbs; and Group 4: grasses. Different letters above each group indicate statistically significant differences across group in species number based on GLMM followed by multiple pairwise comparisons. An asterisk indicates significantly different species numbers between sites for a given group.

In the best GLMM for functional redundancy in the plant communities, the interaction terms functional effect group x scrubland fragmentation and functional effect group x exposure to sea were significant (p <0.001). The combination scrubland fragmentation x Group 3 differed significantly from the combinations with the other groups, which indicated that functional redundancy increased significantly as scrubland fragmentation increases for species in Group 3 (‘short basal annual forbs and perennial forbs’) (Fig. 3). Group 3 exhibited significantly less functional redundancy than did species in Group 1 (‘woody plants’). The combination of exposure to sea x Group 1 differed significantly from the combinations with the other groups, which indicated that functional redundancy in this group was lower at sea-facing sites (Fig. 4).

**Fig 5:**
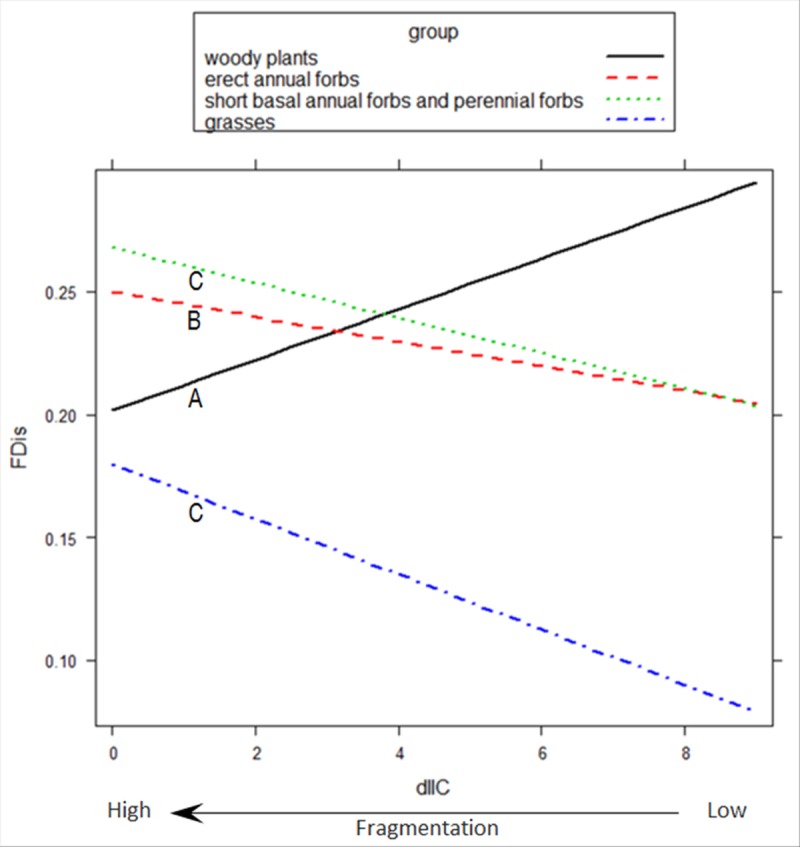
Functional dispersion for plant dispersal of four functional effect groups across a scrubland fragmentation gradient. Fitted values for the optimal Gaussian GLMM model applied to the observed functional dispersion (FDis), weighted by relative abundance of species, for the mechanism of plant dispersal of four functional effect groups in plant communities across a scrubland fragmentation gradient (dIIC) in Cabo de Gata-Níjar Natural Park, Spain. dIIC (here, square-root transformed) is the relative contribution (%) of each scrubland fragment surveyed to overall landscape connectivity. Low dIIC indicates high fragmentation. For clarity, the observed functional dispersion points are omitted. Group 1: woody plants; Group 2: Erect annual forbs; Group 3: short basal annual forbs and perennial forbs; and Group 4: grasses. Different letters below each line indicate statistically significant differences in FDis values with fragmentation across groups based on GLMM followed by multiple pairwise comparisons.

**Fig 6:**
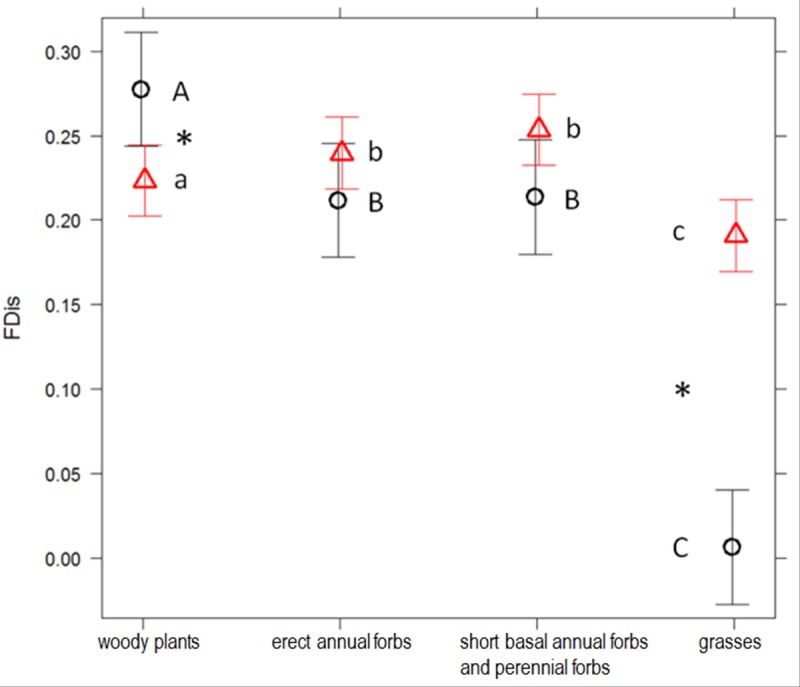
Functional dispersion for plan dispersal of four functional effect groups at non-sea-facing and sea-facing sites. Average functional dispersion (FDis), weighted by relative abundance of species, for the mechanism of plan dispersal of four functional effect groups at non-sea-facing (circles) and sea-facing (triangles) sites in Cabo de Gata-Níjar Natural Park, Spain. Group 1: woody plants; Group 2: Erect annual forbs; Group 3: short basal annual forbs and perennial forbs; and Group 4: grasses. Different letters above each group indicate statistically significant differences across group in FDis values based on GLMM followed by multiple pairwise comparisons. An asterisk indicates significantly different FDis values between sites for a given group.

The best GLMM for response diversity in each functional effect group for the plant dispersal function indicated that the interaction terms functional effect group x scrubland fragmentation, functional effect group x exposure to sea, and functional effect group x distance to sea were significant. Response diversity for the plant dispersal function decreased significantly as scrubland fragmentation increased in Group 1, but it increased in Groups 3 and 4 (p <0.0001; Fig. 5). Response diversity was significantly lower in Group 1 and higher in Group 4 at sea-facing sites (p <0.0001; Fig. 6). As distance to the sea increased, response diversity increased significantly in Group 4, but decreased in Group 2 (p <0.0001).

## Discussion

In the semi-arid Mediterranean scrubland of Cabo de Gata-Níjar Natural Park, Spain, scrubland fragmentation has impacted functional composition (i.e., functional richness and functional dispersion) in the plant communities. Scrubland fragmentation increased the functional richness in the plant communities. Thus, contrary to our predictions, the number of unique trait-value combinations in the plant communities increased as scrubland fragmentation increased. Habitat fragmentation is one of the main causes of biodiversity loss [29], [31], [66], but see [67]; however, in Cabo de Gata-Níjar Natural Park, Alados et al. [68] demonstrated that fragment size and the number of plant species (species richness) were not correlated. In our study, functional richness (measured using 12 traits simultaneously) in the plant communities was higher at the most fragmented sites. This fact might have been related to time-lagged responses of communities to habitat fragmentation [69], or to the well-adapted traits of the plants at fragmented sites such as successional species [45], [51] and species from neighbouring habitats (e.g., agricultural matrix; see [31], [67], [70]). Although functional richness was higher at the most fragmented sites, functional dispersion was lower. Thus, the dispersion (i.e., spread) of trait values among species in the functional trait space was lower at the most fragmented sites (i.e., lower degree of trait dissimilarity among species based on the traits measured). The reduction in functional dispersion in response to an increase in scrubland fragmentation was similar to what occurred in plant communities disturbed by eutrophication [71]. This fact reflected the disappearance of plant trait combinations or the reduction of extreme trait values in the plant community, indicating a reduction in the degree of niche differentiation among the species [15]. Those results suggest that scrubland fragmentation increased the vulnerability of ecosystem functioning in the plant communities because the vegetation in the semi-arid Mediterranean scrubland had low ecological tolerance to habitat fragmentation.

In our study, the functional effect groups, in which species had similar ecological effects on ecosystem functioning, differed in their resilience to scrubland fragmentation. Our analysis showed that, among short basal annual forbs and perennial forbs (Group 3), functional redundancy increased as scrubland fragmentation increased because the number of plant species adapted to disturbed soils and, therefore, probably suited to fragmented habitats increased [35], [45]. Functional redundancy in the other three functional effect groups (‘woody plants’, ‘erect annual forbs’, and ‘grasses’) remained unchanged in the face of an increase in scrubland fragmentation because the number of species within these groups did not vary significantly in response to an increase in scrubland fragmentation. However, changes in plant species composition has occurred (S2 Appendix), which might have detrimental effects on the species sensitive to scrubland fragmentation. In the same study area, Alados *et al*. [68] found that turnover in species composition was associated with scrubland fragmentation, but without a change in species richness. Several plant traits, particularly those related to plant dispersal, were strongly correlated with species composition differentiation [68], which influenced the vulnerability of plant species to an increase in scrubland fragmentation. In our study, an increase in scrubland fragmentation had a negative effect on the response diversity among woody plants; i.e., their responses to scrubland fragmentation were less diverse at the most fragmented sites, as reflected by the low dissimilarity of the values of the traits associated with plant dispersal. Response diversity is an effective proxy of ecosystem resilience because low response diversity is correlated with an increase in the probability that species performing similar functions (i.e., in the same functional group) are lost after a disturbance, because all species are affected similarly, and the function can be lost [1], [5], [6]. The loss of resilience in plant dispersal by ‘woody plants’ in response to scrubland fragmentation might have a negative effect on ecosystem stability and the capacity to recover from ecological disturbances. This might be important because woody plants substantially contribute to the community biomass and, therefore, influence the key ecosystem functions (e.g., energy, carbon, and nitrogen cycles) in the semi-arid Mediterranean scrubland.

Unlike ‘woody plants’, in the functional effect groups ‘short basal annual forbs and perennial forbs’ and ‘grasses’, fragmentation and response diversity were positively correlated, which suggests that these two groups had high resilience to scrubland fragmentation. For example, the dominant grass *Stipa tenaccissima* (the most abundant plant in the study area, 34% of the plant cover) was very abundant at the fragmented sites (S2 Appendix). This tussock is a successful colonizer because it grows clonally by extensively branched rhizomes [72], which confers a competitive advantage to this species in the study area [73–75]. In addition, the species exhibits trypanocarpy and bradyspory mechanisms, which are helpful in reducing seed losses through ant depredation and in increasing seed germination [76], [77].

In the semi-arid areas of SE Spain, *Stipa* steppes are derived from the degradation of woody vegetation [47], [73], [74], [78]. Furthermore, *Stipa* steppes have been favoured by grazing, burning, and harvesting for fibre during decades at the expense of scrubland [45], [78]. Despite its economic importance, *Stipa* steppe is an impoverished vegetation type [78] compared to the semi-arid scrubland, which is mainly characterized by patches that face the harsh arid conditions [79]. Those shrub patches are key components in vegetation structure, production, and dynamics [35], [78] because they have high biological productivity [78], [80], more available niches [52], [78], [81], and high nutrient content (organic C, total N, potential N mineralization [82]). In addition, scrubland supports several ecosystem services such as soil erosion control [83], gas regulation (e.g. carbon sequestration; see [82]), fodder production [84], fuel provision [82], cultural benefits (small game hunting [85]), conservation (endemic plant species [86] and refuge for endangered species [37], [87]) in arid ecosystems. An increase in the fragmentation of scrubland can, however, jeopardize the benefits that woody plants provide to humans because of the low capacities of woody plants to respond to this disturbance, as indicated by the functional responses documented in our study.

Sea-facing areas receive highly humid air because of the inflows of moist marine air driven by sea breezes, which are common around the Mediterranean Sea, and have a marked influence on the weather at coastal areas, and even at sites as far as 300 km inland [88], [89]. In our study, sea-borne humidity affected the plant communities. In response to an increase in fragmentation, the functional dispersion in the plant communities at the dry sites decreased, but remained unchanged at the sites that had high humidity (i.e., sea-facing sites). Exposure to sea did not affect the functional redundancy in the functional effect groups, except ‘woody plants’, which was higher at the dry sites. At those sites, response diversity was higher among ‘woody plants’ than it was among ‘grasses’, which demonstrated how aridity can act as an environmental filter on Mediterranean vegetation by favouring ‘woody plants’ in the semi-arid scrubland [35], [74].

In conclusion, the multidimensional functional approach (i.e., using multiple functional traits) in our study detected changes in the functional composition of the plant communities caused by scrubland fragmentation. In addition, that approach allowed us to infer changes in vegetation resilience to fragmentation in multiple functional effect groups. Identification of the key traits that influence the sensitivity of plants to scrubland fragmentation remains a challenge, however, even though it is fundamental in ecology and conservation biology. As Villéger *et al*. [12] and Laliberté *et al*. [6] indicated, the functional traits for analyses should be chosen carefully and be as directly associated with the ecosystem functions of interest as possible. The multidimensional functional approach helps to increase understanding about the effects of land degradation on ecosystem functioning and provides guidance for the conservation of semi-arid Mediterranean scrubland, particularly, the vulnerable functional effect group ‘woody plants’. Conservation planners can increase the resilience of that group by increasing the connectivity of the scrubland fragments.

## Supporting Information

S1 Appendix Environmental data. Environmental data [exposure to the sea (sites facing or not facing the sea), distance to the sea (km), and scrubland fragmentation measure (dIIC, see text)] of the 18 natural scrubland fragments surveyed in Cabo de Gata-Níjar Natural Park, Spain.
(XLSX)

S2 Appendix Plant species abundance. Plant species abundance (mean ± SD) in the 18 natural scrubland fragments surveyed in Cabo de Gata-Níjar Natural Park, Spain.
(XLSX)

S1 Fig Functional dispersion in plant communities at non-sea-facing and sea-facing sites across a scrubland fragmentation gradient. Fitted values for the optimal Gaussian GLMM model applied to the observed functional dispersion (FDis), weighted by relative abundance of species, in plant communities across a scrubland fragmentation gradient (dIIC) in Cabo de Gata-Níjar Natural Park, Spain. dIIC (here, square-root transformed) is the relative contribution (%) of each scrubland fragment surveyed to overall landscape connectivity. Low dIIC indicates high fragmentation. Solid line indicates non-sea-facing sites and dotted line indicates sea-facing sites. For clarity, observed functional dispersion points are omitted.
(DOCX)

S2 Fig Functional dispersion in plant communities in relation to the distance to the sea. Fitted values (solid line) and 95% confidence band (grey band) for the optimal Gaussian GLMM model applied to the observed functional dispersion (FDis), weighted by relative abundance of species, in plant communities and the distance to the sea in Cabo de Gata-Níjar Natural Park, Spain.
(DOCX)

S1 Table Percentage cover of the plant species. Percentage cover of the plant species in the scrubland of Cabo de Gata-Níjar Natural Park, Spain. Scientific names follow Blanca *et al*. (2009).
(DOCX)

S2 Table Correlations between functional traits. Correlations between functional traits.
(XLSX)

S3 Table Number of levels of each trait within each functional group. Number of levels of each trait within each functional group.
(DOCX)
