# Supplementary material for: Inferring Resilience to Fragmentation-Induced Changes in Plant Communities in a Semi-Arid Mediterranean Ecosystem
Source: PLoS One. 2015 Mar 19;10(3):e0118837. doi: 10.1371/journal.pone.0118837 (PMC4366014; doi:10.1371/journal.pone.0118837)

**S1 Fig.** Fitted values for the optimal Gaussian GLMM model applied to the observed functional dispersion (FDis), weighted by relative abundance of species, in plant communities across a scrubland fragmentation gradient (dIIC) in Cabo de Gata-Níjar Natural Park, Spain. dIIC (here, square-root transformed) is the relative contribution (%) of each scrubland fragment surveyed to overall landscape connectivity. Low dIIC indicates high fragmentation. Solid line indicates non-sea-facing sites and dotted line indicates sea-facing sites. For clarity, observed functional dispersion points are omitted.


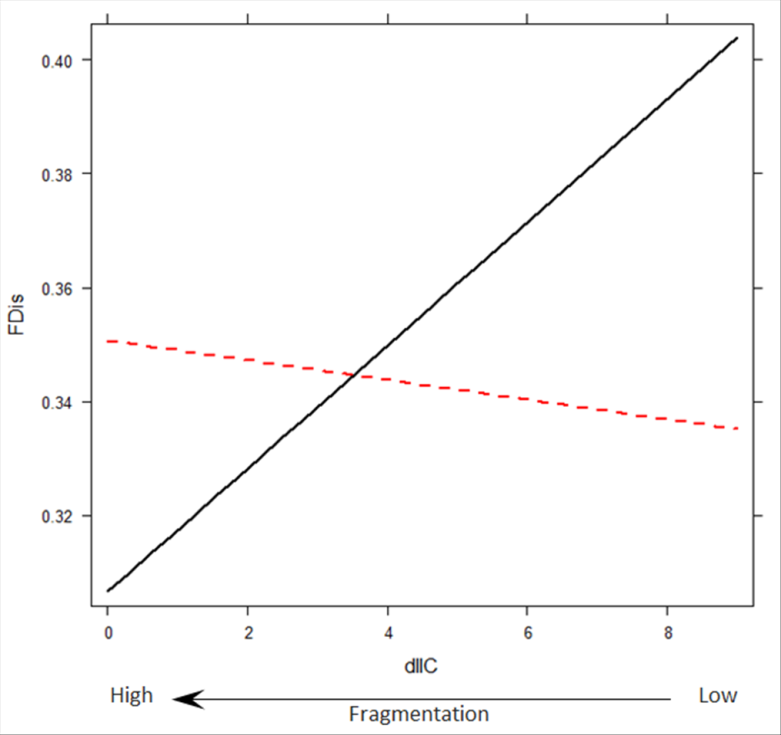

Supplement: S1 Fig — Fitted values for the optimal Gaussian GLMM model applied to the observed functional dispersion (FDis), weighted by relative abundance of species, in plant communities across a scrubland fragmentation gradient (dIIC) in Cabo de Gata-Níjar Natural Park, Spain. dIIC (here, square-root transformed) is the relative contribution (%) of each scrubland fragment surveyed to overall landscape connectivity. Low dIIC indicates high fragmentation. Solid line indicates non-sea-facing sites and dotted line indicates sea-facing sites. For clarity, observed functional dispersion points are omitted. (DOCX) [file pone.0118837.s003.docx]
