# Supplementary material for: Inferring Resilience to Fragmentation-Induced Changes in Plant Communities in a Semi-Arid Mediterranean Ecosystem
Source: PLoS One. 2015 Mar 19;10(3):e0118837. doi: 10.1371/journal.pone.0118837 (PMC4366014; doi:10.1371/journal.pone.0118837)

**S2 Fig.** Fitted values (solid line) and 95% confidence band (grey band) for the optimal Gaussian GLMM model applied to the observed functional dispersion (FDis), weighted by relative abundance of species, in plant communities and the distance to the sea in Cabo de Gata-Níjar Natural Park, Spain.


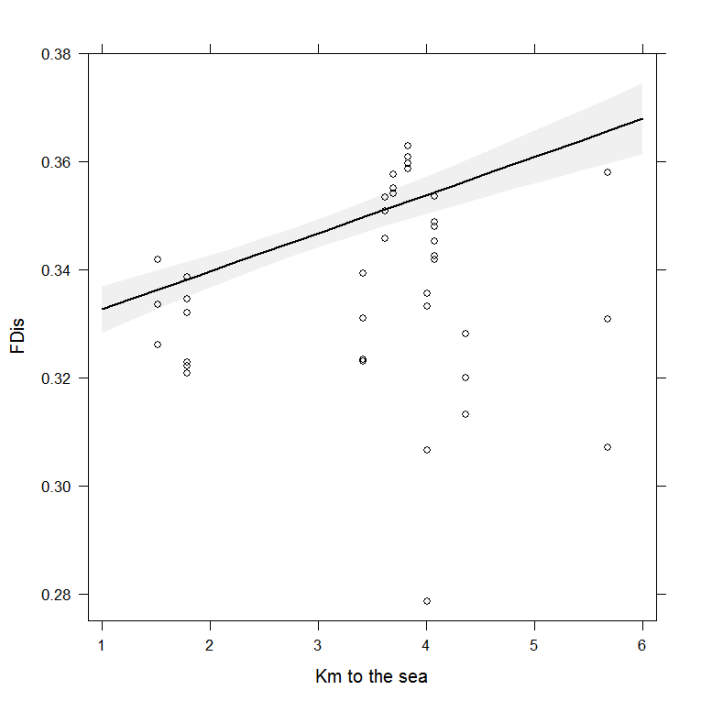

Supplement: S2 Fig — Fitted values (solid line) and 95% confidence band (grey band) for the optimal Gaussian GLMM model applied to the observed functional dispersion (FDis), weighted by relative abundance of species, in plant communities and the distance to the sea in Cabo de Gata-Níjar Natural Park, Spain. (DOCX) [file pone.0118837.s004.docx]
