# Supplementary material for: Inferring Resilience to Fragmentation-Induced Changes in Plant Communities in a Semi-Arid Mediterranean Ecosystem
Source: PLoS One. 2015 Mar 19;10(3):e0118837. doi: 10.1371/journal.pone.0118837 (PMC4366014; doi:10.1371/journal.pone.0118837)
Supplement: S1 Table — Percentage cover of the plant species in the scrubland of Cabo de Gata-Níjar Natural Park, Spain. Scientific names follow Blanca et al. (2009). (DOCX) [file pone.0118837.s005.docx]

**S1 Table.** Percentage cover of the plant species in the scrubland of Cabo de Gata-Níjar Natural Park, Spain. Scientific names follow Blanca *et al.* (2009).

| Species | Plant cover (%) |
| --- | --- |
| *Stipa tenacissima* | 33.80 |
| *Chamaerops humilis* | 8.29 |
| *Brachypodium retursum* | 5.56 |
| *Thymus hyemalis* | 5.40 |
| *Periploca laevigata* | 3.53 |
| *Brachypodium distachyum* | 2.64 |
| *Phlomis purpurea* | 1.97 |
| *Launea lanifera* | 1.91 |
| *Stipa capensis* | 1.82 |
| *Plantago amplexicaulis* | 1.61 |
| *Lavandula multifida* | 1.45 |
| *Ulex parviflorus* | 1.27 |
| *Genista spartoides* | 1.21 |
| *Anthyllis cytisoides* | 1.18 |
| *Dactylis glomerata* | 1.11 |
| *Genista ramosissima* | 0.94 |
| *Linum strictum* | 0.94 |
| *Asparagus horridus* | 0.92 |
| *Helianthemum almeriense* | 0.85 |
| *Teucrium charidemi* | 0.81 |
| *Ballota hirsuta* | 0.78 |
| *Carduus tenuiflorus* | 0.77 |
| *Silene tetraphylla* | 0.70 |
| *Salsola genistoides* | 0.61 |
| *Bromus rubens* | 0.58 |
| *Leontodon longirrostris* | 0.58 |
| *Asteriscus maritimus* | 0.56 |
| *Asphodelus fistulosus* | 0.52 |
| *Fumana thymifolia* | 0.49 |
| *Arenaria montana* | 0.47 |
| *Brassica repanda* | 0.47 |
| *Lygeum spartum* | 0.46 |
| *Stoibrax dichotomum* | 0.46 |
| *Phagnalon saxatile* | 0.44 |
| *Avena barbata* | 0.44 |
| *Asparagus albus* | 0.44 |
| *Cistus albidus* | 0.42 |
| *Artemisia herba-alba* | 0.40 |
| *Convolvulus siculus* | 0.39 |
| *Fagonia cretica* | 0.38 |
| *Sideritis oxteoxylla* | 0.38 |
| *Melica minuta* | 0.38 |
| *Psoralea bituminosa* | 0.36 |
| *Genista umbellata* | 0.34 |
| *Avena sterilis* | 0.32 |
| *Plantago afra* | 0.31 |
| *Convolvulus althaeoides* | 0.29 |
| *Thapsia villosa* | 0.29 |
| *Plantago bellardi* | 0.28 |
| *Arrhenatherum album* | 0.28 |
| *Dianthus charidemi* | 0.27 |
| *Asphodelus tenuifolius* | 0.26 |
| *Sedum sediforme* | 0.25 |
| *Lycium intrincatum* | 0.24 |
| *Euphorbia exigua* | 0.21 |
| *Teucrium pseudochamaepitys* | 0.21 |
| *Paronychia suffruticosa* | 0.20 |
| *Rhamnus lyciodes* | 0.20 |
| *Filago pyramidata* | 0.20 |
| *Echium creticum* | 0.19 |
| *Fumana laevipes* | 0.19 |
| *Launaea arborescens* | 0.18 |
| *Avenula gervaisii* | 0.18 |
| *Hyparrhenia hirta* | 0.17 |
| *Carlina hispanica* | 0.17 |
| *Plantago lagopus* | 0.17 |
| *Olea europaea* | 0.15 |
| *Teucrium eirocephalum* | 0.15 |
| *Cephalaria leucantha* | 0.14 |
| *Hippocrepis ciliata* | 0.13 |
| *Rosmarinus officinalis* | 0.13 |
| *Teucrium lusitanicum* | 0.12 |
| *Sedum album* | 0.12 |
| *Euphorbia segetalis* | 0.12 |
| *Anagallis arvensis* | 0.11 |
| *Ruta agustifolia* | 0.10 |
| *Eryngium campestre* | 0.10 |
| *Limonium lobatum* | 0.09 |
| *Lobularia maritima* | 0.09 |
| *Sonchus tenerrimus* | 0.07 |
| *Lavatera maritima* | 0.07 |
| *Pallenis spinosa* | 0.07 |
| *Klasea flavescens* | 0.07 |
| *Lavandula stoechas* | 0.07 |
| *Phlomis lychnitis* | 0.07 |
| *Atractylis cancellata* | 0.07 |
| *Satureja intincata* | 0.06 |
| *Euphorbia serrata* | 0.06 |
| *Helichrysum stoechas* | 0.05 |
| *Limonium sinuatum* | 0.05 |
| *Globularia alypum* | 0.05 |
| *Polygala rupestris Pourr.* | 0.05 |
| *Reichardia picroides* | 0.05 |
| *Crepis vesicaria* | 0.03 |
