# Supplementary material for: Inferring Resilience to Fragmentation-Induced Changes in Plant Communities in a Semi-Arid Mediterranean Ecosystem
Source: PLoS One. 2015 Mar 19;10(3):e0118837. doi: 10.1371/journal.pone.0118837 (PMC4366014; doi:10.1371/journal.pone.0118837)
Supplement: S3 Table — Number of levels of each trait within each functional group. (DOCX) [file pone.0118837.s007.docx]

**S3 Table.** Number of levels of each trait within each functional group.

| Functional effect group | Growth form | Main growth form | Life cycle | Clonality | Plant height | Seed mass | Seed number | Potential spatial dispersal | Alternative spatial dispersal | Biological type | Pollination syndrome | Plant architecture |
| --- | --- | --- | --- | --- | --- | --- | --- | --- | --- | --- | --- | --- |
| 1 | Dwarf shrubs (n=33) | Woody species (n=44) | Perennial (n=44) | Absent (n=39) | <10 cm (n=1) | 0.01-0.099 mg (n=12) | 0-250 (n=7) | Developed spatial dispersal by abiotic vectors (n=16) | Absent (n=42) | Fruticose chamaephyte (n=10) | Anemophily (n=1) | Champagnat † (n=1) |
|  | Erect leafy (n=1) |  |  | Present (n=5) | 11-29 cm (n=7) | 0.1-0.999 mg (n=12) | 251-500 (n=3) | Developed spatial dispersal by biotic vectors (n=4) | Present (n=2) | Pulvinular chamaephyte (n=1) | Entomophily (n=43) | Corner ** (n=1) |
|  | Leafless (n=4) |  |  |  | 30-59 cm (n=20) | 1-9.999 mg (n=15) | 501-1000 (n=6) | Restricted spatial dispersal (n=24) |  | Creeping chamaephyte (n=1) |  | Corner † (n=1) |
|  | Palmoid (n=1) |  |  |  | 60-99 cm (n=10) | 10-99,999 mg (n=3) | 1001-2500 (n=14) |  |  | Succulent chamaephyte (n=2) |  | Holttum ** (n=2) |
|  | Short basal (n=1) |  |  |  | 1-3 m (n=6) | 100-1000 mg (n=2) | 2501-5000 (n=12) |  |  | Sufruticose chamaephyte (n=13) |  | Holttum † (n=1) |
|  | Shrub (n=3) |  |  |  |  |  | >5001 (n=2) |  |  | Escapiform hemicryptophyte (n=1) |  | Leeuwenberg † (n=5) |
|  | Tree (n=1) |  |  |  |  |  |  |  |  | Genistoid nano-phanerophyte (n=4) |  | Rauh * (n=3) |
|  |  |  |  |  |  |  |  |  |  | Evergreen nano-phanerophyte (n=11) |  | Scarrone *** (n=15) |
|  |  |  |  |  |  |  |  |  |  | Pulvinular nano-phanerophyte (n=1) |  | Scarrone + (n=11) |
|  |  |  |  |  |  |  |  |  |  |  |  | Scarrone † (n=1) |
|  |  |  |  |  |  |  |  |  |  |  |  | Missing data (n=3) |
| 2 | Erect leafy (n=12) | Herb (n=12) | Annual (n=12) | Absent (n=12) | 11-29 cm (n=7) | 0.01-0.099 mg (n=4) | 0-250 (n=8) | Developed spatial dispersal by abiotic vectors (n=3) | Absent (n=10) | Erect therophyte (n=11) | Anemophily (n=1) |  |
|  |  |  |  |  | 30-59 cm (n=2) | 0.1-0.999 mg (n=5) | 251-500 (n=1) | Restricted spatial dispersal (n=9) | Present (n=2) | Creeping therophyte (n=1) | Entomophily (n=11) |  |
|  |  |  |  |  | 60-99 cm (n=3) | 1-9.999 mg (n=3) | 501-1000 (n=3) |  |  |  |  |  |
| 3 | Erect leafy (n=10) | Herb (n=25) | Annual (n=6) | Absent (n=19) | <10 cm (n=1) | 0.01-0.099 mg (n=4) | 0-250 (n=12) | Developed spatial dispersal by abiotic vectors (n=15) | Absent (n=20) | Caespitose chamaephyte (n=1) | Anemophily (n=3) |  |
|  | Short basal (n=15) |  | Perennial (n=19) | Present (n=6) | 11-29 cm (n=4) | 0.1-0.999 mg (n=7) | 251-500 (n=7) | Developed spatial dispersal by biotic vectors (n=2) | Present (n=5) | Creeping chamaephyte (n=1) | Entomophily (n=22) |  |
|  |  |  |  |  | 30-59 cm (n=9) | 1-9.999 mg (n=12) | 501-1000 (n=4) | Restricted spatial dispersal (n=8) |  | Sufruticose chamaephyte (n=3) |  |  |
|  |  |  |  |  | 60-99 cm (n=11) | 10-99,999 mg (n=2) | 1001-2500 (n=2) |  |  | Rhizomatous geophyte (n=1) |  |  |
|  |  |  |  |  |  |  |  |  |  | Erect hemicryptophyte (n=4) |  |  |
|  |  |  |  |  |  |  |  |  |  | Escapiform hemicryptophyte (n=3) |  |  |
|  |  |  |  |  |  |  |  |  |  | Creeping hemicryptophyte (n=1) |  |  |
|  |  |  |  |  |  |  |  |  |  | Rosulate hemicryptophyte (n=2) |  |  |
|  |  |  |  |  |  |  |  |  |  | Climber nano-phanerophyte (n=2) |  |  |
|  |  |  |  |  |  |  |  |  |  | Erect therophyte (n=2) |  |  |
|  |  |  |  |  |  |  |  |  |  | Rosulate therophyte (n=5) |  |  |
| 4 | Non tussock grass (n=11) | Grass (n=13) | Annual (n=6) | Absent (n=6) | 11-29 cm (n=2) | 0.1-0.999 mg (n=3) | 0-250 (n=5) | Developed spatial dispersal by abiotic vectors (n=8) | Absent (n=4) | Caespitose chamaephyte (n=1) | Anemophily (n=13) |  |
|  | Tussock grass (n=2) |  | Perennial (n=7) | Present (n=7) | 30-59 cm (n=3) | 1-9.999 mg (n=9) | 251-500 (n=1) | Developed spatial dispersal by biotic vectors (n=1) | Present (n=9) | Caespitose hemicryptophyte (n=7) |  |  |
|  |  |  |  |  | 60-99 cm (n=8) | 10-99,999 mg (n=1) | 501-1000 (n=6) | Restricted spatial dispersal (n=4) |  | Caespitose therophyte (n=2) |  |  |
|  |  |  |  |  |  |  | 1001-2500 (n=1) |  |  | Erect therophyte (n=3) |  |  |
